# The Preferences of Transgender and Nonbinary People for Virtual Health Care After the COVID-19 Pandemic in Canada: Cross-sectional Study

**DOI:** 10.2196/40989

**Published:** 2022-10-26

**Authors:** Jose M Navarro, Ayden I Scheim, Greta R Bauer

**Affiliations:** 1 Department of Epidemiology and Biostatistics Schulich School of Medicine & Dentistry Western University London, ON Canada; 2 Department of Epidemiology and Biostatistics Dornsife School of Public Health Drexel University Philadelphia, PA United States

**Keywords:** virtual care, telemedicine, telehealth, eHealth, transgender, gender identity, COVID-19, gender-affirming care, older adult, mental health, chronic condition, social support

## Abstract

**Background:**

Virtual health care use has dramatically increased in response to the COVID-19 pandemic, raising the question of its potential role after the pandemic. For transgender (trans) and nonbinary (TNB) people, virtual care is promising because it may expand access to appropriate health care providers. However, emerging research indicates potential disparities in virtual care access related to sociodemographic, health, and social factors. There is a paucity of research on the factors affecting patient preferences for virtual versus in-person care, particularly in TNB communities.

**Objective:**

This study aimed to identify the sociodemographic, health, and social factors associated with postpandemic virtual care preferences in TNB communities.

**Methods:**

The 2020 Trans PULSE Canada COVID survey examined the health, social, and economic impacts of the COVID-19 pandemic among 820 TNB participants who previously completed the prepandemic 2019 Trans PULSE Canada survey (n=2783). Data were weighted to the demographics of the 2019 sample. Chi-square tests were used to compare postpandemic preferences for virtual versus in-person care across sociodemographic, health, and social characteristics. Participants provided open-text responses explaining their preferences, which were used to contextualize quantitative findings.

**Results:**

Among 812 participants who indicated whether they would prefer virtual or in-person care after the pandemic, a weighted 32.7% (n=275) would prefer virtual care and 67.3% (n=537) would prefer in-person care. Preference for in-person over virtual care was associated with being in the 14-19 (49/56, weighted 85.0%), 50-64 (51/62, weighted 80.0%), and ≥65 (9/10, weighted 90.7%) age groups (*χ*^2^_5_=19.0; *P=*.002). Preference for virtual over in-person care was associated with having a chronic health condition (125/317, weighted 37.7% versus 150/495, weighted 29.9%; *χ*^2^_1_=4.7; *P*=.03) and having probable anxiety (229/645, weighted 34.7% versus 46/167, weighted 25.7%; *χ*^2^_1_=4.3; *P*=.04). Among participants with romantic partners, preferences varied based on the partner’s level of support for gender identity or expression (*χ*^2^_3_=13.3; *P*=.004). Participants with moderately supportive partners were more likely than participants with very supportive partners to prefer in-person care (36/43, weighted 85.1% versus 275/445, weighted 62.3%). Care preferences did not vary significantly based on the indicators of socioeconomic status. Open-text responses showed that multiple factors often interacted to influence participant preferences, and that some factors, such as having a chronic condition, simultaneously led some participants to prefer virtual care and others to prefer in-person care.

**Conclusions:**

TNB people may have differential interest in virtual care based on factors including age, chronic and mental health conditions, and gender-unsupportive home environments. Future research examining virtual care preferences would benefit from mixed methods intersectional approaches across these factors, to explore complexity in the barriers and facilitators of virtual care access and quality. These observed differences support flexibility with options to choose between in-person and virtual health care to meet TNB patients’ specific health needs.

## Introduction

Virtual care, also known as telemedicine or telehealth, has historically been underused due to concerns about quality of care, insufficient regulatory frameworks, lack of technological infrastructure, and its limited inclusion in public health insurance programs in countries with publicly funded health systems like Canada [1-3]. In 2020, the COVID-19 pandemic forced health care institutions to adapt services for remote delivery and drove Canadian provincial and territorial governments to rapidly strengthen public health insurance coverage for virtual care [4-6].

Moving forward from the pandemic, transgender (trans) and nonbinary (TNB) communities may benefit from increased access to virtual care [7]. People who are trans have a gender identity that differs from the sex they were assigned at birth [8,9]. People who are nonbinary, who may or may not identify as trans, have gender identities beyond woman or man [9]. TNB people often struggle to find health care providers who are clinically and culturally competent to address TNB health issues [8], and clinics that specialize in gender-affirming care (eg, hormone therapy) tend to be limited to major urban centers [7,9]. TNB people also frequently report experiences of stigma and discrimination while traveling to and accessing health care, leading to care avoidance and unmet health care needs [8,10]. Virtual care could help address these concerns by allowing TNB people to access a broader array of both general and gender-affirming health services and practitioners, regardless of their place of residence [1,7,11]. Travel costs and lost income would also be reduced by virtual care delivery [1,7,11,12]. Current gender-affirming virtual care options like Connect-Clinic [13] in Ontario show promise in this regard, but they are still limited, with demand far exceeding supply [7]. Moreover, gender-affirming care is only one subset of the health care needed by TNB patients.

Despite its promise, the adoption of virtual care must be approached with caution as current research on its benefits and drawbacks is relatively limited and provides mixed results [1,3,6,14]. Access to virtual care may be particularly limited for TNB people who lack a reliable internet connection, lack safe confidential spaces where they can access virtual care, or lack the digital literacy needed to effectively use online health services [11,12,14-17]. Although public funding of virtual care has expanded in Canada, it is still limited [6]. For instance, at the time of writing, most jurisdiction codes only cover care delivered via video call and not via secure text messaging [3], and some provinces place a cap on the number of virtual appointments that physicians can bill to public health insurance [6]. Along with parallel for-profit models of telemedicine, this limited coverage could translate into socioeconomic disparities in virtual care access for TNB people [2-4,15].

With the goal of informing postpandemic virtual care practice and policies, this paper draws on data from a national survey of TNB people in Canada conducted during the COVID-19 pandemic to examine how preferences indicated for postpandemic in-person versus virtual care varied based on sociodemographic, health-related, and social characteristics. We also drew on qualitative data from open-text fields to elucidate the reasons for participants’ preferences.

## Methods

### Participants and Procedures

Administered from September 21 to October 20, 2020, the Trans PULSE Canada COVID survey collected national data on the health, economic, and social impacts of the COVID-19 pandemic on TNB people in Canada [18]. Participants were recruited from a list of 1187 TNB Canadian residents aged ≥14 years (as of 2019) who consented for recontact after completing the 2019 Trans PULSE Canada survey. Details of the methods for the 2019 study have been published previously. In brief, participants were recruited via convenience sampling and completed the survey on paper, by telephone, or online [19]. Eligible participants for the 2020 COVID survey were contacted via their preferred communication method among email, telephone, text, or letter mail and directed to a webpage with further explanation of the study. Of the 1187 people contacted, 820 (69.1%) completed the 2020 survey. Consent was implied by survey completion. All questionnaires were self-administered online in English or French through REDCap [20], although participants were also offered the option of a mailed questionnaire and of receiving accessibility supports such as translation. Survey questions were pretested for clarity, and participants could skip any question they did not wish to answer. Participants provided separate (optional) consent for the publication of quotes from open-text fields. A CAD $20 (US $15) gift card honorarium was offered to each participant who completed the 2020 COVID survey.

### Ethics Approval

The Research Ethics Boards at Western University, Drexel University, and Wilfrid Laurier University approved the 2020 Trans PULSE Canada COVID survey (Western University: project ID 116072; Drexel University: protocol number 2005007801; Wilfrid Laurier University: REB number 6557).

### Measures

#### Primary Outcome

Virtual care was defined for participants as “health care or medical advice delivered via phone call, video call, or text message.” Preference for virtual versus in-person health care was indicated by the response to the following question: “In general, would you prefer virtual over in-person care when COVID-19 is no longer an issue?” Those who responded “yes” were categorized as preferring virtual care and those who responded “no” were categorized as preferring in-person care. Participants were also asked to explain the reasons for their preference in an open-response text box.

#### Sociodemographic, Health-Related, and Social Factors

Sociodemographic characteristics included age, gender, racialization, indigeneity, province or territory, rurality, and indicators of socioeconomic status (annual income, employment status, low-income household, and housing stability). Racialized participants were those who self-identified or were perceived or treated as people of color in Canada. Those living in a town or municipality with a population less than 10,000 were categorized as rural, based on postal code. The definition of a low-income household was based on Statistics Canada’s low-income measure [21], and examples of unstable housing included living in a shelter, motel, or car.

Health-related factors included self-identification as disabled, chronic conditions, virtual care experiences, mental health conditions, and gender-affirming care status. Participants were identified as having a chronic condition if they indicated that they had chronic pain, a chronic illness, or a chronic health condition. Participants were asked the following 4 questions related to virtual care experiences: (1) whether they had accessed virtual care since March 12, 2020, the start of the COVID-19 pandemic; (2) the type of virtual care received (physical, mental, or other including gender-affirming care); (3) the platform over which they received virtual care (phone call, video call, texting, or other including email); and (4) whether they had avoided virtual care due to their TNB identity since the start of the COVID-19 pandemic. Current mental health was assessed using validated scales. Anxiety symptoms were measured using the Overall Anxiety Severity and Impairment Scale (OASIS) [22]. Participants responded to 5 items, each with 5 options (coded 0-4 with a possible score range of 0-20), indicating the relative frequency or intensity of their anxiety symptoms in the past week. Summed scores of 8 or above indicated probable anxiety [22]. Depressive symptoms were measured with the 10-item abridged Center for Epidemiologic Studies Depression Scale (CES-D-10) [23]. Each item had 4 response options (coded 0-3 with a possible score range of 0-30), with higher summed scores reflecting a greater frequency of depressive symptomatology. A cutoff score of 10 or above indicated potential clinical depression [23]. Participants were also asked the extent to which they had received or were considering gender-affirming medical care, which was defined in the survey as including “puberty blockers, gender-affirming hormones, surgeries, or body modifications.”

Participants responded to questions assessing their social environment and relationships, that is, whether they had experienced intimate partner violence since August 2019, how supportive their partner(s) and parent(s)/guardian(s) were of their gender identity or expression, and whether they were concerned about family stress from confinement and violence at home during the pandemic.

### Analysis

As less than 1% of COVID survey participants (8 of 820) did not respond to the question on postpandemic preferences for virtual care, these participants were excluded from the analysis. Responses to the 2020 COVID survey were weighted to match the demographic profile of the full 2019 sample on characteristics like age, ethnoracial background, and socioeconomic status, using a raking algorithm. Weights were used in case loss to follow-up between the 2019 and COVID surveys was nonrandom and to allow for better comparability between the pre-COVID and COVID samples. Rao-Scott chi-square tests with α=.05 were performed on weighted data to compare preferences for virtual versus in-person care after the pandemic across sociodemographic, health, and social factors. All quantitative analyses were carried out in SAS 9.4. software (SAS Institute Inc).

Similar to a sequential explanatory design [24], direct quotations from participants’ open-text responses are included in the results, with indications of the cited participant’s age, gender, and province to contextualize quantitative findings. Themes corresponding to the statistically significant quantitative results were identified. Open-text responses explaining virtual care preferences were sorted into these themes.

## Results

### Virtual Care Preferences and Identified Themes

Of 812 participants, a weighted 32.7% (n=275) said they would prefer virtual care after the COVID-19 pandemic, while 67.3% (n=537) would prefer in-person care. Most participants (746/812, 91.9%) provided an explanation for their virtual care preference in the open-response question. Based on the quantitative findings that follow, the following broad themes were identified, into which the open-text responses were sorted: age, disability and chronic conditions, mental health, social environment, and the logistics of care access (eg, convenience and technological literacy). After further examination of open-text responses, we identified discrimination and stigma as another prominent theme not present in the quantitative results. Table 1 presents the unweighted sociodemographic characteristics of the 812 participants included in the analysis.

**Table 1:** Sociodemographic characteristics of the analytic sample.

Characteristic		Total sample (N=812), n (%)^a^
**Age (years)**		
	14-19	56 (6.9)
	20-24	147 (18.2)
	25-34	306 (38.0)
	35-49	225 (27.9)
	50-64	62 (7.7)
	≥65	10 (1.2)
**Gender**		
	Woman or girl	200 (24.7)
	Man or boy	198 (24.4)
	Indigenous or cultural gender identity	18 (2.2)
	Nonbinary or similar	395 (48.7)
**Racialization**		
	Yes	108 (13.3)
	No	702 (86.7)
**Indigenous in Canada**		
	Yes	59 (7.3)
	No	750 (92.7)
**Immigration status**		
	Newcomer (past 5 years)	23 (2.8)
	Immigrant (nonnewcomer)	91 (11.2)
	Born in Canada	698 (86.0)
**Province of residence**		
	Alberta	151 (18.6)
	Atlantic^b^	38 (4.7)
	British Columbia	176 (21.7)
	Manitoba	27 (3.3)
	Newfoundland and Labrador	6 (0.7)
	Ontario	305 (37.6)
	Quebec	81 (10.0)
	Saskatchewan	26 (3.2)
	Territories^c^	1 (0.1)
**Rural**		
	Yes	47 (5.8)
	No	762 (94.2)
**Personal annual income (CAD$^d^; age≥16 years)**		
	None	142 (17.8)
	<$14,999	214 (26.8)
	$15,000-$29,999	160 (20.1)
	$30,000-$49,999	124 (15.5)
	$50,000-$79,999	94 (11.8)
	≥$80,000	64 (8.0)
**Education**		
	Less than high school	44 (5.4)
	High school diploma	62 (7.6)
	Some college or university	206 (25.4)
	College or university degree	354 (43.6)
	Graduate/professional degree	145 (17.9)
**Employment situation (age ≥25 years)**		
	Permanent full-time	220 (37.0)
	Employed, not permanent full-time	206 (34.6)
	Not employed or on leave	136 (22.9)
	Not employed and student or retired	33 (5.5)
**Low-income household(past year; age≥16 years**)		
	Yes	310 (41.1)
	No	444 (58.9)
**Housing stability**		
	Stable	806 (99.3)
	Unstable	6 (0.7)
**Disability identity**		
	Yes	217 (26.7)
	No	595 (73.3)

^a^Unweighted frequencies and proportions are reported.

^b^Including New Brunswick, Nova Scotia, and Prince Edward Island.

^c^Including Northwest Territories, Nunavut, and Yukon.

^d^A currency exchange rate of CAD $1=US $0.75 is applicable.

### Sociodemographic Differences

Age was associated with care preferences (*χ*^2^_5_=19.0; *P=*.002), with a larger proportion of participants aged 14-19 years (49/56, weighted 85.0%), 50-64 years (51/62, weighted 80.0%), and ≥65 years (9/10, weighted 90.7%) preferring in-person care after the pandemic compared with participants in other age groups (Table 2). Gender identity was also associated with care preference (*χ*^2^_3_=11.2; *P*=.01), with participants self-identifying with an Indigenous or culturally specific gender minority identity being more likely to prefer virtual care than those identifying as women, men, or nonbinary, although no significant difference in virtual care preference was observed by indigenous identity (*χ*^2^_1_=0.02; *P=*.90). No significant associations were identified between care preferences and the various indicators of socioeconomic status.

**Table 2:** Preference for virtual care versus in-person care by sociodemographic characteristics in Trans PULSE Canada COVID survey participants.

Characteristic			Prefers virtual care (N=275)				Prefers in-person care (N=537)					*P* value^a^
			Value, n (%)^b^		95% CI^b^		Value, n (%)^b^		95% CI^b^			
**Age (years)**											.002	
	14-19	7 (15.0)		4.6-25.4		49 (85.0)		74.6-95.4				
	20-24	52 (34.1)		25.9-42.2		95 (65.9)		57.8-74.1				
	25-34	114 (37.1)		31.3-42.9		192 (62.9)		57.1-68.7				
	35-49	90 (38.7)		31.7-45.6		135 (61.3)		54.4-68.3				
	50-64	11 (20.0)		8.4-31.5		51 (80.0)		68.5-91.6				
	≥65	1 (9.3)		0.0-26.9		9 (90.7)		73.1-100.0				
**Gender**											.01	
	Woman or girl	61 (29.1)		22.4-35.9		139 (70.9)		64.1-77.6				
	Man or boy	56 (26.1)		19.6-32.6		142 (73.9)		67.4-80.4				
	Indigenous or cultural gender identity	10 (55.4)		31.3-79.5		8 (44.6)		20.5-68.7				
	Nonbinary or similar	148 (37.0)		31.9-42.2		247 (63.0)		57.8-68.1				
**Racialization**											.69	
	Yes	38 (34.5)		24.9-44.2		70 (65.5)		55.8-75.1				
	No	236 (32.4)		28.7-36.2		466 (67.6)		63.8-71.3				
**Indigenous in Canada**											.90	
	Yes	22 (33.6)		21.0-46.3		37 (66.4)		53.7-79.0				
	No	253 (32.8)		29.2-36.4		497 (67.2)		63.6-70.8				
**Province of residence**											—^c^	
	Alberta	49 (31.4)		23.5-39.2		102 (68.6)		60.8-76.5				
	Atlantic^d^	9 (24.1)		9.9-38.2		29 (75.9)		61.8-90.1				
	British Columbia	70 (40.1)		32.3-47.8		106 (59.9)		52.2-67.7				
	Manitoba	9 (27.1)		10.3-43.8		18 (72.9)		56.2-89.7				
	Newfoundland and Labrador	4 (73.7)		38.9-100.0		2 (26.3)		0.0-61.1				
	Ontario	96 (31.0)		25.4-36.5		209 (69.0)		63.5-74.6				
	Quebec	28 (30.6)		20.0-41.1		53 (69.4)		58.9-80.0				
	Saskatchewan	9 (31.3)		12.3-50.2		17 (68.8)		49.8-87.7				
	Territories^e^	1 (100.0)		100.0-100.0		0 (0.0)		0.0-0.0				
**Rural**											.12	
	Yes	20 (43.8)		28.5-59.1		27 (56.2)		40.9-71.5				
	No	254 (32.1)		28.5-35.6		508 (67.9)		64.4-71.5				
**Personal annual income (CAD$^f^; age≥16 years)**											.08	
	None	50 (34.9)		26.3-43.4		92 (65.1)		56.6-73.7				
	<$14,999	78 (34.0)		27.2-40.7		136 (66.0)		59.3-72.8				
	$15,000-$29,999	46 (26.9)		19.7-34.2		114 (73.1)		65.8-80.3				
;	$30,000-$49,999	42 (34.7)		25.6-43.8		82 (65.3)		56.2-74.4				
	$50,000-$79,999	42 (45.6)		34.9-56.4		52 (54.4)		43.6-65.1				
	≥$80,000	16 (24.2)		12.9-35.5		48 (75.8)		64.5-87.1				
**Employment situation (age ≥25 years)**											.40	
	Permanent full-time	87 (39.3)		32.4-46.3		133 (60.7)		53.7-67.6				
	Employed, not permanent full-time	69 (32.9)		26.0-39.9		137 (67.1)		60.1-74.0				
	Not employed or on leave	48 (34.5)		25.7-43.3		88 (65.5)		56.7-74.3				
	Not employed and student or retired	9 (25.4)		9.2-41.6		24 (74.6)		58.4-90.8				
**Low-income household(past year; age≥16 years**)											.23	
	Yes	114 (35.5)		29.8-41.3		196 (64.5)		58.7-70.2				
	No	145 (31.1)		26.5-35.7		299 (68.9)		64.3-73.5				
**Housing stability**											.46	
	Stable	273 (32.6)		29.1-36.1		533 (67.4)		63.9-70.9				
	Unstable	2 (48.1)		4.3-92.0		4 (51.9)		8.0-95.7				

^a^Comparing care preferences across sociodemographic characteristics using the Rao-Scott chi-square test.

^b^Proportions are weighted to the sociodemographics of the prepandemic Trans PULSE Canada sample.

^c^*P* value was not available owing to small cell sizes.

^d^Including New Brunswick, Nova Scotia, and Prince Edward Island.

^e^Including Northwest Territories, Nunavut, and Yukon.

^f^A currency exchange rate of CAD $1=US $0.75 is applicable.

### Disability and Chronic Conditions

Preference for virtual care was more common in participants with chronic conditions (125/317, weighted 37.7% versus 150/495, weighted 29.9%; *χ*^2^_1_=4.7; *P*=.03) (Table 3). Although no significant differences in care preferences were found based on self-identification as disabled (*χ*^2^_1_=0.43; *P*=.51), 1 participant who preferred virtual over in-person care noted:

With COVID, things are going virtual and it is so helpful. I am more connected now, and people make more of an effort for virtual visits as well. This is the kind of access people living with my disability need.

Nonbinary or similar, aged 25-34 years, living in Ontario

At the same time, others cited their chronic health conditions as a reason to prefer in-person care:

I think virtual visits would be great long term for things like prescription refills or ordering things that don't require a physical inspection. But with chronic health concerns I feel being visually seen in person for a checkup throughout the year is vital to my staying healthy and functional.

Man, aged 25-34 years, living in Ontario

These sentiments echo another common theme in participant open-text responses—that preferences for virtual versus in-person care were contingent on the specific type of care being sought. Numerous participants noted a preference for virtual care for appointments they deemed as not requiring in-person treatment (like prescription refills), and a preference for in-person care when they felt that aspects like physical examination were necessary.

Open-text responses also showed that the examined sociodemographic, health-related, and social factors often interacted to affect virtual care preferences. For instance, 1 participant discussed how their chronic condition exacerbated geographic barriers to in-person care:

Taking public transit to and from an appointment when I'm already not feeling well can take a substantial amount of energy. Having to do this over and over again for ongoing investigation into my symptoms takes even more. I desperately need this energy for basic caretaking of myself… I shouldn't have to make myself sicker to access health care.

Nonbinary or similar, aged 25-34 years, living in Ontario

**Table 3:** Disability and chronic conditions, virtual care experiences, mental health factors, and social environment of Trans PULSE Canada COVID cohort participants preferring virtual versus in-person care.

Characteristic				Prefers virtual care (N=275)				Prefers in-person care (N=537)							*P* value^a^		
				Value, n (%)^b^		95% CI^b^		Value, n (%)^b^		95% CI^b^							
**Disability identity**													.51				
	Yes		78 (34.8)		28.0-41.5		139 (65.2)				58.5-72.0						
	No		197 (32.1)		28.1-36.2		398 (67.9)				63.8-71.9						
**Chronic conditions**										.			.03				
	Yes		125 (37.7)		32.0-43.5		192 (62.3)				56.5-68.0						
	No		150 (29.9)		25.5-34.2		345 (70.1)				65.8-74.5						
**Virtual care access since March 12, 2020**													<.001				
		Yes	200 (38.4)		33.8-43.0		299 (61.6)		57.0-66.2								
		No	75 (24.5)		19.3-29.6		238 (75.5)		70.4-80.7								
**Type of virtual care^c^**																	
		Physical health care	173 (39.3)		34.3-44.4		250 (60.7)		55.6-65.7			.42					
		Mental health care	118 (39.6)		33.4-45.8		164 (60.4)		54.2-66.6			.56					
		Other	5 (57.8)		25.4-90.2		5 (42.2)		9.8-74.6			.22					
**Virtual care platform^c^**																	
		Phone call	176 (38.0)		33.1-42.9		264 (62.0)		57.1-66.9			.67					
		Video call	113 (42.0)		35.4-48.6		139 (58.0)		51.4-64.6			.14					
		Texting/SMS text messaging	19 (32.7)		18.9-46.5		36 (67.3)		53.5-81.1			.41					
		Other	1 (100.0)		100.0-100.0		0 (0.0)		0.0-0.0			—^d^					
**Virtual care avoidance because of trans/nonbinary identity since March 12, 2020**													.14				
		Yes	48 (38.9)		29.7-48.2		76 (61.1)		51.8-70.3								
		No	227 (31.6)		27.9-35.4		461 (68.4)		64.6-72.1								
**Anxiety**													.04				
		Probable anxiety (OASIS^e^ ≥8)	229 (34.7)		30.7-38.7		416 (65.3)		61.3-69.3								
		No probable anxiety (OASIS <8)	46 (25.7)		18.6-32.8		121 (74.3)		67.2-81.4								
**Depression**													.06				
		Probable depression (CES-D-10^f^ ≥10)	242 (34.2)		30.3-38.0		445 (65.8)		62.0-69.7								
		No probable depression (CES-D-10 <10)	33 (25.1)		17.0-33.2		92 (74.9)		66.8-83.0								
**Gender-affirming medical care status**													.52				
		Had all needed care	84 (28.6)		22.8-34.4		198 (71.4)		65.6-77.2								
		In the process of completing	101 (33.6)		27.9-39.4		187 (66.4)		60.6-72.1								
		Planning, but not begun	32 (38.4)		27.4-49.3		54 (61.6)		50.7-72.6								
		Unsure if going to seek care	27 (33.2)		22.0-44.4		46 (66.8)		55.6-78.0								
		Not planning	31 (35.4)		24.7-46.1		52 (64.6)		53.9-75.3								
**Experienced intimate partner violence (since August 2019)**																	
		Yes	38 (26.2)		18.5-33.9		93 (73.8)		66.1-81.5			.10					
		No	234 (33.8)		30.0-37.7		439 (66.2)		62.3-70.0								
**Spouse/partner support of gender identity or expression^g^**													.004				
		Very supportive	170 (37.7)		32.8-42.6		275 (62.3)		57.4-67.2								
		Not very or somewhat supportive	7 (14.9)		4.3-25.6		36 (85.1)		74.4-95.7								
		Not at all supportive	5 (69.1)		36.2-100.0		3 (30.9)		0.0-63.8								
		Does not know about gender identity/expression	1 (20.0)		0.0-58.8		2 (80.0)		41.2-100.0								
**Parent/guardian support of gender identity or expression^g^**													.06				
		Very supportive	78 (29.0)		23.0-35.1		177 (71.0)		64.9-77.0								
		Not very or somewhat supportive	122 (34.0)		28.7-39.3		227 (66.0)		60.7-71.3								
		Not at all supportive	35 (47.2)		35.0-59.4		40 (52.8)		40.6-65.0								
		Does not know about gender identity/expression	27 (31.9)		20.9-42.8		54 (68.1)		57.2-79.1								
**Concerned about family stress from confinement due to COVID-19**													.09				
		Extremely or very	111 (35.4)		29.6-41.2		194 (64.6)		58.8-70.4								
		Somewhat	74 (27.2)		21.4-33.1		192 (72.8)		66.9-78.6								
		Not at all	89 (35.5)		29.0-42.0		150 (64.5)		58.0-71.0								
**Concerned about violence at home during COVID-19**													.98				
		Extremely or very	9 (31.9)		13.1-50.7		16 (68.1)		49.3-86.9								
		Somewhat	18 (31.6)		18.1-45.1		36 (68.4)		54.9-81.9								
		Not at all	248 (32.9)		29.2-36.5		485 (67.1)		63.5-70.8								

^a^Comparing care preferences across experiences with virtual care, mental health factors, and social environment factors using the Rao-Scott chi-square test.

^b^Proportions are weighted to the sociodemographics of the prepandemic Trans PULSE Canada sample.

^c^Among those who received virtual care since March 12, 2020 (n=499).

^d^*P* value was not available owing to small cell sizes.

^e^OASIS: Overall Anxiety Severity and Impairment Scale.

^f^CES-D-10: 10-item abridged Center for Epidemiologic Studies Depression Scale.

^g^Results reported only for participants who indicated that these questions were applicable to them.

### Virtual Care Experiences

Among participants who accessed virtual care since the pandemic (n=499, weighted 59.4%), 38.4% (weighted, 200/499) indicated a preference for virtual care after the pandemic, a significantly greater proportion than participants who did not access virtual care (75/313, weighted 24.5%; *χ*^2^_1_=14.4; *P*<.001) (Table 3). Among those who accessed virtual care, postpandemic preferences did not vary depending on whether they accessed it for physical (*χ*^2^_1_=0.66; *P*=0.42) or mental (*χ*^2^_1_=0.35; *P*=0.56) health care, or whether they received care via phone (*χ*^2^_1_=0.18; *P*=0.67), video call (*χ*^2^_1_=2.2; *P*=0.14), or texting (*χ*^2^_1_=0.68; *P*=0.41). Multiple participants mentioned that they received gender-affirming care, specifically hormone therapy, via virtual means, and that email was another platform through which they received care, which was not listed in our survey. Postpandemic virtual care preferences did not vary based on whether participants had avoided virtual care during the pandemic due to their TNB identities (*χ*^2^_1_=2.2; *P*=.14).

### Mental Health Factors

A majority (n=645, weighted 78.3%) of the sample had probable anxiety (indicated by OASIS scores≥8), and participants with probable anxiety were more likely to prefer virtual care after the pandemic than participants without (229/645, weighted 34.7% versus 46/167, weighted 25.7%; *χ*^2^_1_=4.3; *P*=.04) (Table 3). Similarly, 84.3% (weighted, n=687) of the sample had CES-D-10 scores≥10, indicating clinically significant depressive symptomatology. A higher proportion of participants reaching this cutoff preferred virtual care, although this difference only approached statistical significance (242/687, weighted 34.2% versus 33/125, weighted 25.1%; *χ*^2^_1_=3.5; *P*=.06).

Consistent with our quantitative results, mental health conditions acted as barriers to accessing in-person treatment for some participants, with 1 participant saying:

It is sometimes difficult to leave the house or go to new environments without assistance given [my] anxiety and mental health issues - having the option to do virtual [care] makes health care more accessible.

Nonbinary, aged 20-24 years, living in British Columbia

At the same time, mental health concerns had different implications for care among other participants. One participant stated the following:

Phone conversations make me anxious, and I will avoid [them] just because it's going to be a phone conversation. Video calls are worse than phone calls. I feel like a lot of physical health issues need to be seen or felt, and mental health issues are better conveyed in person.

Nonbinary, aged 25-34 years, living in British Columbia

While not captured in our quantitative results, several participants also cited gender dysphoria (discomfort arising when one’s physical characteristics do not align with their gender identity [6]) as a reason for preferring in-person care. Some participants explained that they experienced gender dysphoria while attending appointments via video call because they had to see themselves on screen. With regard to telephone appointments, 1 participant expressed the following:

My voice is higher than I would like, so talking over the phone makes me dysphoric about how the professional on the other line sees me based on my voice.

Nonbinary, aged 20-24 years, living in Saskatchewan

### Social Environment

Most TNB participants with a spouse or romantic partner had one who was very supportive of their gender identity or expression (445/499, weighted 89.0%) (Table 3). Spouse or partner support was associated with care preference (*χ*^2^_3_=13.3; *P*=.004), with participants having moderately supportive partners being more likely to prefer in-person care than participants having very supportive partners (36/43, weighted 85.1% versus 275/445, weighted 62.3%). No significant differences depending on having experienced intimate partner violence were found (*χ*^2^_1_=2.7; *P*=.10).

Participants with very supportive parents or guardians more frequently indicated a preference for in-person care (177/255, weighted 71.0%) compared with other participants, although there were no statistically significant differences (*χ*^2^_3_=7.5; *P*=.06). However, multiple participants, particularly youth, mentioned that a lack of parental support was a reason to prefer in-person care. One participant made the following statement:

I live with my transphobic parents and wouldn't feel comfortable having medical appointments with them nearby.

Nonbinary, aged 14-19 years, living in Ontario

Privacy issues with other family members and roommates were also cited in open-text responses as reasons to avoid virtual care.

Participants preferring virtual care also attributed their preference to previously experienced discrimination in health care settings. One participant explained:

As a trans person, healthcare settings are a place where I've experienced a lot of abuse and oppression. My providers now are mostly good, but the setting is triggering… I don't miss not having to expose myself to that.

Woman, aged 35-49 years, living in British Columbia

In contrast, numerous participants stated that they were more likely to be misgendered (referred to as the wrong gender [9]) in virtual care settings, justifying a preference for in-person care. One participant made the following statement:

To be honest, it has been tough being misgendered constantly in my home over virtual meetings. I'd prefer in-person care elsewhere, so home becomes a safer space again, with less misgendering.

Nonbinary or similar, aged 25-34 years, living in British Columbia

Other participants explicitly noted that their TNB identity was irrelevant to their virtual care preferences.

## Discussion

This paper identifies factors that may influence postpandemic preferences for virtual versus in-person care among TNB people in Canada. While most participants preferred in-person care, around 1 in 3 (weighted 32.7%) indicated a postpandemic preference for virtual care. Lack of access to virtual care during the pandemic was associated with postpandemic preference for in-person care, highlighting the importance of identifying and addressing the challenges that certain populations disproportionately face while attempting to access telemedicine. Participants who were aged 14-19 or ≥50 years were more likely to prefer in-person care over virtual care compared with other age groups. Other research has shown a lower level of digital literacy among older adults as a factor contributing to preference for in-person care [11,15-17]. Given that all participants in this study completed the survey online and therefore likely had substantial digital literacy, our finding of a similar age-related preference suggests that additional factors may be at play. Consistent with previous findings [16], chronic conditions and anxiety symptoms were associated with preferring virtual care over in-person care, suggesting that telemedicine offers a promising alternative for those whose health conditions prevent them from safely and comfortably attending in-person appointments. Older adults, with a higher prevalence of chronic conditions compared with the general population [25], may therefore particularly stand to benefit from telemedicine.

Previous research predicted that unsupportive home environments could compromise the privacy and sense of safety necessary to access virtual care [11], and our results in general support this with regard to a lack of support for gender identity or expression in the home. Having gender-unsupportive romantic partners was associated with preference for in-person care. Associations with gender support from parents or guardians were less clear, which may be because relevance is age dependent. Privacy issues may in part explain why participants preferring virtual care were less likely to be adolescents (age 14-19 years), who may have no option but to live with family or roommates, including those who are unsupportive (or who do not know). However, we did not measure whether our participants were actually living with their romantic partners, or their parents or guardians at the time of participation. Regardless, these privacy concerns highlight how a move to virtual care could disadvantage those in unsafe or unsupportive home environments, especially younger people who may not have the freedom or financial means to live on their own.

It is important to note that our quantitative results only captured the overall preferences of the sample, and closer examination of qualitative results showed heterogeneity. For example, some participants reported that anxiety prevented them from accessing in-person care, whereas others had more anxiety surrounding virtual care. While this heterogeneity was expected, the broad operationalizations used for some of our variables may have obscured salient differences. For instance, participants were categorized based on identity as disabled, which does not capture the wide diversity in disability experiences. Accordingly, some participants with physical disabilities cited barriers to accessing in-person care, whereas others reported that disabilities like autism made virtual care less accessible. Similar variability was found for mental health and other chronic conditions. Future studies would benefit from examining these conditions with more nuanced and detailed categorization, for instance, by distinguishing between the types of disabilities, chronic conditions, and mental health conditions.

Another limitation of this study was that, because the survey focused broadly on the COVID-19 experiences of TNB people, only a few questions assessed virtual care experiences. Thus, some key factors like the ability to find a private space to access virtual care were not directly measured. Further, the survey’s online administration mode may have introduced selection bias favoring those with preferences for virtual care. In general, although this study used the largest national sample of TNB people in Canada during the COVID-19 pandemic, the results should be interpreted with caution and are not generalizable to all TNB communities given that participants were a subset of a convenience sample.

A strength of this study was its use of qualitative responses to elucidate and elaborate upon quantitative results. While open-ended survey items generally do not produce the rich data of a true qualitative study, these responses highlighted how the factors under study could not be discussed in isolation from one another. Rather, our limited quotes suggest that they act together, for example, the ways a chronic health condition intersects with geographic barriers to require repeated public transit trips that then exacerbate the health condition. This suggests that future research should draw on an intersectionality theoretical framework to explore these processes of interaction and how they may relate to social power [26]. Future qualitative research would contribute to a deeper understanding of the processes through which virtual care preferences and access play out across intersections of gender, race, disability, chronic disease, age, and socioeconomic status, and a mixed methods intersectional approach would aid in the development of more nuanced and well-rounded virtual care policies and programs.

Some participants expressed through open-text responses that their TNB identity was relevant to their virtual care preferences, mentioning gender dysphoria, transphobia in healthcare, or misgendering. Similar proportions of participants who did and did not avoid virtual care during the pandemic due to their TNB identity reported postpandemic preference for virtual care. This finding suggests that those who avoided virtual care due to their TNB identity, for instance, due to anticipated discrimination [8], may similarly avoid in-person care. However, many participants did not mention their gender, with some explicitly stating that their preference was unrelated to their TNB identity. Because many of our quantitative findings and open-text responses were related to non-TNB-specific factors, they may be applicable to the broader population.

The heterogeneity within our results suggests that flexibility in choice regarding modality of care delivery may best support the diverse needs of patients, particularly those in TNB communities. For example, because some participants justified preferences for in-person care based on anxiety, chronic conditions, and parental support, while others used these factors to justify virtual care preferences, practitioners should be prepared to deliver care via virtual or in-person means, if feasible. Flexibility should also be afforded in terms of the mode of virtual care delivery. Some TNB participants experienced dysphoria when seeing themselves on a video call, but others may have been more concerned with practitioners misgendering them based on how their voice sounded over telephone appointments.

Depending on the province or territory in Canada where physicians practice, they may face limitations on the types and modes of services they can bill to public health insurance, which may influence the care options that they are willing or able to provide [3,6]. In certain jurisdictions, physicians have daily caps on the number of virtual appointments they can bill to public health insurance [6]. While previous studies found that patients often want care delivered over secure text messaging, when possible, few jurisdictions in Canada offer publicly funded texting services [3]. Only certain types of care are publicly insured, with, for instance, walk-in appointments not being covered in Nova Scotia [6]. Additionally, many changes made in the Canadian virtual care policy in response to the COVID-19 pandemic are still temporary [6]. To improve care quality and access, policymakers and funders may consider implementing permanent funding schemes that cover a wider range of services and reward physicians similarly for the same services delivered via different modes.

Although virtual care may be valuable to address existing disparities in care access for TNB-specific and nonspecific services, both virtual and in-person care options will remain important after the pandemic. Researchers and care providers should continue to identify populations for whom virtual care is particularly beneficial, identify the barriers that may prevent them from accessing it, and propose and appraise interventions designed to overcome these disparities.

## Abbreviations

CES-D-10: 10-item abridged Center for Epidemiologic Studies Depression Scale
OASIS: Overall Anxiety Severity and Impairment Scale
TNB: transgender (trans) and nonbinary
